# Oxidative Stress Modulation and Glutathione System Response During a 10-Day Multi-Stressor Field Training

**DOI:** 10.3390/jfmk10020166

**Published:** 2025-05-10

**Authors:** Liāna Pļaviņa, Edgars Edelmers

**Affiliations:** 1 Department of Morphology, Rīga Stradiņš University, LV-1010 Riga, Latvia; 2 National Arm Forces, National Defence Academy of Latvia, LV-1014 Riga, Latvia; 3 Medical Education Technology Centre, Rīga Stradiņš University, LV-1067 Riga, Latvia; edgars.edelmers@rsu.lv

**Keywords:** endurance training, oxidative stress, antioxidant system

## Abstract

**Objectives:** To evaluate how a 10-day multi-stressor field-training course—combining high physical and psycho-emotional demands, caloric restriction, and severe sleep deprivation—affects systemic oxidative/antioxidative status and biomarkers of nucleic-acid and skeletal-muscle damage in trained military cadets. **Methods:** Seventy-five healthy cadets (8 women, 67 men; 22–34 y) completed the course. Standardised operational rations (700–800 kcal day^−^¹) and two 20 min tactical naps per 24 h were enforced. Pre- and post-course venous blood was collected after an overnight fast. Plasma superoxide-dismutase activity (SOD), reduced and oxidised glutathione (GSH, GSSG), malondialdehyde (MDA), and hydrogen peroxide (H₂O₂) were quantified by colourimetric/fluorometric assays; 8-hydroxy-2-deoxyguanosine (8-OHdG) and myoglobin were measured by ELISA. The oxidative-stress index (OSI) was calculated as GSSG·GSH^−^¹. Within-subject differences were assessed with Wilcoxon signed-rank tests; associations between biomarker changes were explored by Spearman correlation. **Results:** After training, GSH (+175%, *p* < 0.001) and GSSG (+32%, *p* < 0.001) rose significantly, whereas SOD (−19%, *p* = 0.002), H₂O₂ (−20%, *p* = 0.015), MDA (−50%, *p* < 0.001), 8-OHdG (−23%, *p* < 0.001), and OSI (−47%, *p* < 0.001) declined. Myoglobin remained unchanged (*p* = 0.603). Reductions in MDA correlated inversely with increases in GSSG (rₛ = −0.25, *p* = 0.041), while H₂O₂ changes correlated positively with GSSG (rₛ = 0.25, *p* = 0.046), indicating a glutathione-driven adaptive response. **Conclusions:** Ten consecutive days of vigorous, calorie- and sleep-restricted field training elicited a favourable redox adaptation characterised by enhanced glutathione-mediated antioxidant capacity and lower circulating oxidant concentrations, without evidence of DNA or skeletal-muscle damage. The data suggest that, in physically prepared individuals, prolonged multi-stressor exposure can strengthen endogenous antioxidant defences rather than precipitate oxidative injury.

## 1. Introduction

The metabolic processes in cells are associated with the formation of reactive oxygen species (ROS) and the peroxidation of lipids and proteins, which can be mitigated by components of the antioxidant system [1,2]. Intensive physical training increases metabolism and ROS formation, primarily in muscle [3], which can result in short- and long-term oxidative stress (OS) and cellular damage [2,4,5,6]. In contrast, moderate intensity exercise has systemic health-promoting effects [7,8].

The 10-day training in a multi-stressor environment with various intensive physical activities, sleep, and deprivation of energy intake has complex effects on the participants [9]. The potential adverse effects of long-term training on health include a decrease in body mass index [10], an increase in oxidants in peripheral blood samples, and a decrease in antioxidant response [6,11,12], which can contribute to oxidative stress (OS) and overtraining [11]. An effective adaptation to intensive physical training can eliminate OS by stimulating the antioxidative system intracellularly [1,2]. This process occurs intensively in skeletal muscles [13]. The season of the year [13], the degree of physical exertion [14], the level of physical performance [15], and the type of sports [16] are among factors affecting adaptation to the intensive physical load and OS. Studies on long-term intensive training in athletes and other professionals [9,11,12,16,17,18,19,20] showed inconsistent changes in the components of the antioxidative system and oxidants in the peripheral blood during physical activities.

According to Lushchak [2], the detection of levels of oxidants, components of the antioxidative system, should be assessed simultaneously with markers of cell damage representing the biological effect of OS. It can help control the intensity of OS and simultaneously allow control of the negative effects of physical load [2]. Therefore, our study aimed to investigate the effect of 10-day multi-stressor training on the antioxidative system, combined with evaluating levels of oxidants and markers of nucleic acid and muscle cell damage.

## 2. Materials and Methods

### 2.1. Study Design

The study protocol was reviewed and approved by the National Defence Academy of Latvia and the Rīga Stradiņš University Research Ethics Committee (protocol No 6-3/49, 12 December 2018). All procedures complied with the Declaration of Helsinki and written informed consent was obtained from all participants.

Nutritional and sleep-restriction profile: For the entirety of the 10-day course cadets received one standardised operational ration providing 700–800 kcal day (≈55% CHO, 25% fat, 20% protein) with no supplementary snacks. Water intake was not restricted and no participant exhibited clinical or self-reported signs of dehydration.

Sleep deprivation regimen: rest was restricted to two strictly supervised 20 min tactical naps per 24 h, resulting in an average sleep opportunity of 0.7 ± 0.1 h day.

Operational-security note: A fully detailed day-by-day training schematic cannot be published; the descriptive summary represents the maximum disclosure permitted by the Latvian National Armed Forces.

The present work sought to characterise within-subject redox adaptations rather than make causal inferences versus an inactive comparator, and therefore we did not recruit a separate control group. All cadets experienced the identical physical, psychological, tactical, nutritional, and sleep-restriction stimuli described below, allowing us to attribute observed biomarker fluctuations to the multi-stressor course with reasonable confidence within this homogeneous cohort. Eight women met the same entrance fitness standards and completed the course shoulder-to-shoulder with their male peers. Their small representation (11% of the cohort) precluded powered sex-stratified analyses; nevertheless, no practical difficulties or aberrant responses were noted. Future studies will aim to enrol larger, sex-balanced samples or apply stratified statistics to delineate possible sex-specific oxidative stress responses.

The Field Training Course is an accredited component of the Military Leadership curriculum at the National Defence Academy of Latvia. It immerses the cadets in a continuous field environment (24 h day) designed to elicit concurrent physical, cognitive, and psycho-emotional stress.

Physical workload was divided as follows: daily trek distance 14–23 km with 20–25 kg equipment; aerobic conditioning, callisthenics, live-fire marksmanship, casualty evacuation drills, and obstacle-course work were alternated. The intensity progressed from moderate (rating perceived exertion, RPE ≈ 13; heart rate zones 60–69% HR_max_) on day 1 to high (RPE 16; 80–85% HR_max_) on day 5, before decreasing to moderate by day 10. The total cumulative distance travelled was 185.5 km.

None of the cadets engaged in organised competitive sport during the semester before enrolment; all conditioning derived from the Academy’s compulsory programme (three 90 min callisthenics/endurance sessions plus one 10 km loaded run weekly). MET values for each field-exercise task were estimated from the 2022 Compendium of Physical Activities and cross-checked against mean heart rate (%HR_max_) and session-RPE recordings. Marching with 20–25 kg at 4–5 km h^−^¹ corresponded to 6.0–7.5 METs, obstacle-course work to 8–10 METs, and live-fire marksmanship to 4 METs. Daily, weighted averages were as follows:

- Day 1–2 5.0 ± 0.9 METs (moderate, 3–6 METs);
- Day 3–5 8.3 ± 1.1 METs (vigorous, >6 METs);
- Day 6–10 5.4 ± 1.0 METs (upper-moderate).

Cumulatively, 61% of work time was performed at >5 METs. This confirms that the multi-stressor course predominantly imposed vigorous metabolic demands—an exposure known to influence redox balance differently from moderate-intensity exercise.

Psychoemotional stressors included tactical decision making under time pressure, oppositional force scenarios, and graded leadership evaluations. Stress induction and monitoring were standardised by the Latvian Armed Forces, and instructors maintained a fixed scenario script throughout all training intakes.

Sleep was deliberately restricted to two 20 min tactical naps per 24 h, for an averaged sleep opportunity of 0.7 ± 0.1 h·day^−1^. The participants remained continuously outdoors and bivouacked in full kit.

The diet consisted of one 700 to 800 kcal military ration per day (55% carbohydrate, 25% fat, 20% protein). Ad libitum water intake was encouraged (fin canteen availability; ambient temperature 8–20 °C).

The study sample included 75 previously trained cadets of both genders (8 women and 67 males) aged from 21 to 34 years (mean age was 24.9 ± 3.6 years). Before the 10-day training, all participants underwent a medical examination to determine their health status.

The schedule for the 10-day-long multi-stressor field training included a wide range of physical activities (aerobic, strength, conditioning, agility, and specific skills training) with equipment (20–25 kg) organised in the field environment in the summertime with temperature from +8 °C to +20 °C, with an average of +15 °C (data from the local weather service). Participants during a 10-day-long training get over the distance of 185.5 km. The average level of physical activities increased gradually from moderate on the first day to high on the fifth day and decreased to moderate on the tenth day. Participants were deprived of sleep and spent all their time in outdoor activities. They also had dietary limitations (one food intake daily, 700–800 kcal). The water intake was not restricted. The height varied from 164 to 195 cm (mean height was 181 ± 7 cm). Their BMI decreased significantly during the training course from 24.7 ± 2.6 to 24.0 ± 2.4, z = −6.69, *p* < 0.001.

**Figure 1:**
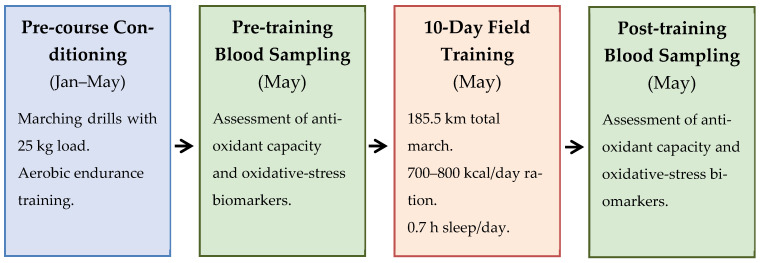
Study timeline.

Figure 1 provides a schematic overview of the study timeline, detailing the pre-course conditioning, pre- and post-training blood-sampling timepoints, and the 10-day multi-stressor field exercise.

### 2.2. Evaluation of Oxidative Stress Parameters

Plasma preparation followed the high-speed modified protocol published by Erel [21,22]. The modified centrifugation at 13,000× *g* for 15 min at 4 °C ensures platelet-free plasma suitable for redox analyses.

Assay catalogue information:

- Myoglobin ELISA Kit (Cat.# MAK120; Sigma-Aldrich, Burlington, VT, USA);
- Superoxide Dismutase Assay Kit (Cat.# 19160-1KT-F; Sigma-Aldrich, USA);
- Total Antioxidant Capacity Assay Kit (Cat.# MAK187; Sigma-Aldrich, USA);
- Glutathione Quantification Kit (Cat.# CS0260; Sigma-Aldrich, USA);
- Fluorometric Hydrogen Peroxide Assay Kit (Cat.# MAK165; Sigma-Aldrich, USA);
- Lipid Peroxidation (MDA) Assay Kit (Cat.# MAK085; Sigma-Aldrich, USA);
- 8-Hydroxy-2′-deoxyguanosine ELISA Kit (Cat.# ab201734; Abcam, Cambridge, UK).

Calculation of oxidative-stress index (OSI): Consistent with Erel [21,22], OSI (arbitrary units) was calculated as the ratio of oxidised glutathione concentrations (GSSG) to reduced glutathione concentrations (OSI = GSSG · GSH^−1^).

Analytical performance characteristics supplied by the manufacturers indicated detection limits of 0.01 opt. d. U. for colorimetric kits and 0.05 ng·mL^−1^ for ELISA-based assays, with intra- and intraassay coefficients of variation < 7%.

Biomarker rationale:

1. Myoglobin is released from damaged skeletal-muscle fibres and therefore tracks exercise-induced sarcolemmal disruption.
2. 8-Hydroxy-2′-deoxyguanosine (8-OHdG) is generated by hydroxyl-radical attack on guanine and serves as a sensitive marker of oxidative DNA injury.
3. Hydrogen peroxide (H_2_O_2_) represents a diffusible, relatively stable non-radical ROS capable of propagating redox signalling and oxidative damage.
4. Malondialdehyde (MDA) is a terminal aldehyde product of polyunsaturated-fatty-acid peroxidation and reflects lipid oxidative injury.
5. Superoxide dismutase (SOD) activity indexes the first-line enzymatic defence against superoxide anion.
6. Reduced (GSH) and oxidised (GSSG) glutathione jointly reflect the principal low-molecular-weight thiol buffer; their ratio (GSSG·GSH^−1^) is reported as the oxidative-stress (OS) index.

All commercial kits exhibited intra-assay and inter-assay coefficients of variation < 7% and analytical sensitivities of 0.01 opt. d. U. (colorimetric assays) or 0.05 ng·mL^−1^ (ELISAs) as reported by the manufacturers.

To assess oxidants and antioxidants, peripheral blood samples were collected in tubes with EDTA in the morning between 7 and 9 o’clock before and after a 10-day-long training course following an overnight fast. Blood samples were centrifuged at 13,000 rpm and +4 °C for 15 min, and plasma was separated and stored before the detection at −80 °C.

As markers of the antioxidative system, superoxide dismutase (SOD) activity (Sigma-Aldrich, USA) and reduced glutathione (GSH) (Sigma-Aldrich, USA) were detected by colorimetric method accordantly the manufacturer’s instructions. Hydrogen peroxide (H_2_O_2_) and malondialdehyde (MDA) were measured by using the Fluorometric Hydrogen Peroxide Assay Kit (Sigma-Aldrich, USA) and the Lipid Peroxidation (MDA) Assay Kit (Sigma-Aldrich, USA) for the assessment of oxidants. Oxidised glutathione (GSSG) was detected using the Glutathione Quantification kit (Sigma-Aldrich, USA). The oxidative stress index (OS index) was calculated as a GSSG/GSH ratio. The results were presented in optical density units (opt. d. U.); absorbance was measured at 450 nm.

### 2.3. Evaluation of Markers of Cell Damage

A marker of muscle damage, myoglobin, was detected in the plasma by using the Myoglobin ELISA kit (Sigma-Aldrich, USA). A marker of nucleic acid damage—8-hydroxy-2-deoxyguanosine (8-OHdG)—was measured using an 8-hydroxy-2-deoxyguanosine ELISA kit.

### 2.4. Statistical Analysis

Nonparametric statistics were chosen because Shapiro–Wilk tests indicated that several biomarker distributions deviated from normality (*p* < 0.05). Consequently, within-subject pre/post differences were assessed using the Wilcoxon signed rank test. Monotonic associations between delta values were evaluated with Spearman’s rank order correlation, which does not assume linearity or normality.

Statistical analyses were performed using the IBM SPSS 22.0 programme. Changes in markers were evaluated using the Wilcoxon test for repeated measures. The relationships between the differences between the first and second measures were assessed using Spearman’s rank correlation test.

## 3. Results

Comparing pre- to post-exercise 8-OHdG concentrations confirmed a significant reduction (*p* < 0.001), whereas paired myoglobin values did not differ (*p* = 0.603), supporting the absence of overt DNA or skeletal-muscle damage despite the imposed stressors.

Plasma 8-OHdG declined significantly (median change −0.45 ng·mL^−1^, *p* < 0.001), whereas myoglobin remained statistically unaltered (median change +0.73 ng·mL^−1^, *p* = 0.603), corroborating the absence of overt DNA or muscular damage despite the imposed load.

Table 1 presents oxidative stress, antioxidative system parameters, and cell damage markers before and after the 10-day-long training. All parameters except for myoglobin demonstrated a significant shift after the training programme.

**Figure 2:**
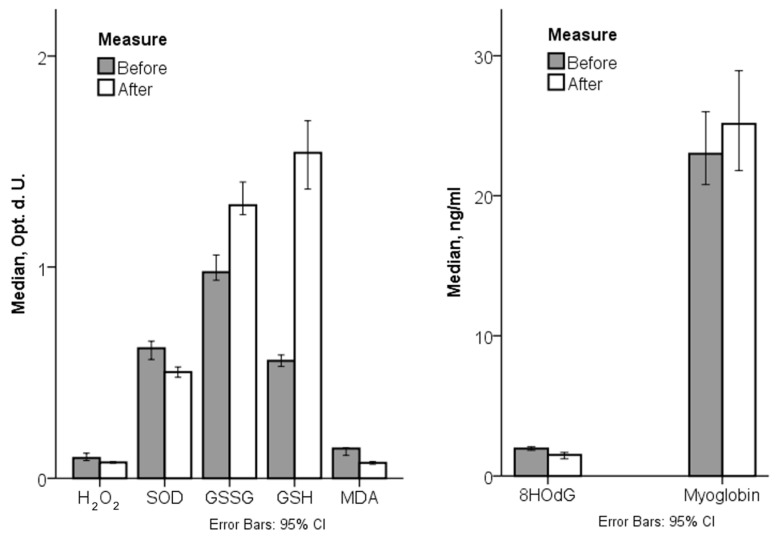
Observed oxidants, antioxidants, and cell damage markers before and after the training programme: (**left**) H_2_O_2_—hydrogen peroxide; SOD—superoxide dismutase activity; GSSG—oxidative glutathione; GSH—reduced glutathione; and MDA—malondialdehyde in opt. d. U.—optical density units; (**right**) 8-OHdG—8-hydroxy-2-deoxyguanosines and myoglobin in ng/mL; CI—confidence interval.

Effects of the training included an increase in GSSGH by 32% and GSH by 175% and a decrease in H_2_O_2_, SOD, 8-OHdG, and MDA levels (Figure 2).

A more intensive decrease was for MDA (50%). H_2_O_2_ level decreased by 20%, SOD activity by 19%, and 8-OHdG by 23%. Simultaneously, the OS index also decreased after the training course by 47%.

Testing of relationships among the differences between the first and second measures revealed a significant negative correlation between changes in MDA and GSSG, rs = −0.25, *p* = 0.041. In addition, the change in H_2_O_2_ was positively associated with a change in GSSG, rs = 0.25, *p* = 0.046.

## 4. Discussion

This study revealed significant changes in components of the oxidative and antioxidative systems after 10-day-long multi-stressor training. Regarding antioxidants, SOD activity decreased after the training course, pointing to the possible development of OS [2]. In contrast, the level of GSH level increased more intensively than the level of GSSG, and the OS index decreased after training. Regarding oxidants, the levels of H_2_O_2_ and MDA in plasma decreased after long-term training. We also did not observe an increase in markers of nucleic acid and muscle damage.

The decrease in oxidants and OS index without an increase in SOD activity partially is in line with the previous study [13], which investigated the dynamic of H_2_O_2_ and MDA simultaneously with the total antioxidant capacity (TAC), SOD, and catalase (CAT) activity in serum after strenuous cycling in the heat. Based on this study [13], the decreasing OS index and oxidants in serum without upregulation of serum TAC, SOD, and CAT in trained athletes was explained by the summertime and partial acclimatization of trained athletes to the heat that could affect the decreasing of oxidants in plasma after training at the myo-cellular level [13]. In addition to the study by Keller et al. (2022), our study revealed an upregulation of glutathione system (GSH and GSSG) simultaneously with the reduction in oxidants in plasma [13]. The levels of H_2_O_2_ and lipid peroxides, such as MDA, can be reduced by direct interaction with GSH, which leads to the formation of GSSG [2]. In our study, correlating changes in MDA and GSSG support increased GSSG after reducing MDA. In addition, related changes in H_2_O_2_ and GSSG also suggest that activity-related oxidative stress stimulates the glutathione system. The decreasing MDA to a lower than the pre-training level was also in line with Jówko et al. (2018) when MDA was measured after 36 h of survival training [6]. The revealed changes in oxidants and antioxidants can be defined as helpful in preventing psychological stress and musculoskeletal injuries associated with training because of the reduction in ROS [3,5,14,23,24].

Long-time training can lead to overtraining [11], which can be assessed by a change in the OS index [11,17]. Despite long-term training in our participants, the decrease in the OS index most likely excludes overtraining in the group. The specificity of the 10-day training with the reduced physical load after the fifth day can add to the decrease in the OS index. Therefore, the measurements of oxidants and antioxidants at the maximal load should be included in further study. Additionally, the decrease in the OS index can be associated with the variation in baseline OS markers [20] and the level of physical performance [15]. In our study, we did not assess the level of physical performance of participants, which is one of the study’s limitations. Although direct physiological monitoring (e.g., heart-rate telemetry) was precluded by operational constraints, the documented cumulative march distance (185.5 km), imposed caloric deficit (~–3500 kcal day^−1^), and severe sleep restriction collectively confirm that participants experienced a pronounced psychophysical stress load.

Myoglobin functions as an intramuscular oxygen reservoir and leaks into the circulation when sarcolemmal integrity is compromised. Basal plasma values in healthy adults are typically <25 ng mL^−1^, while strenuous, unaccustomed exercise can raise concentrations to 150 ng mL^−1^ in non-athletes and >800 ng mL^−1^ in elite endurance competitors. In the present cohort, the pre-exercise median was 23.0 ng mL^−1^ and remained statistically unchanged after the 10-day field course (Δ + 0.73 ng mL^−1^; *p* = 0.603). The absence of a post-training surge—despite cumulative march distance of 185.5 km, severe caloric restriction, and sleep deprivation—suggests that the applied load did not exceed the participants’ myofibrillar repair capacity. Although sampling occurred ~12 h after the final exercise bout (potentially missing an earlier transient peak), the stability of myoglobin supports the conclusion that overt muscle damage was minimal, and that prior conditioning conferred effective protection against exercise-induced rhabdomyolysis.

8-Hydroxy-2′-deoxyguanosine (8-OHdG) is a sensitive surrogate of oxidative modification of guanine bases and therefore a sentinel of systemic DNA oxidation. Contrary to the rise often reported after exhaustive or novel exercise stimuli, circulating 8-OHdG fell by a median of 0.45 ng mL^−1^ (*p* < 0.001) over the training period. This downward shift, paralleled by simultaneous declines in hydrogen peroxide and malondialdehyde, indicates that net oxidative pressure on genomic material was attenuated rather than exacerbated. The finding is congruent with the substantial up-regulation of reduced glutathione and the 47% drop in the glutathione-based oxidative-stress index, implying that ROS generation was adequately counter-balanced—principally through glutathione-dependent pathways—despite the multi-stressor environment.

Taken together, the combination of an unaltered myoglobin profile and a reduced 8-OHdG concentration argues against the development of moderate-to-severe oxidative stress; within Lushchak’s framework, the response is best categorised as mild, adaptive oxidative stress. Nonetheless, the within-subject, non-controlled design, the largely male sample, and the single post-intervention timepoint temper causal inference. Circadian rhythm, seasonal variation, and individual recovery kinetics could each modulate biomarker availability. Future studies incorporating serial sampling, sex-balanced cohorts, and an inactive comparator will be required to confirm whether the observed biomarker constellation truly reflects an enhanced redox resilience induced by repeated field exercise.

Study limitations, in particular, the quasiexperimental nature of the design and absence of a contemporaneous control group preclude causal inference; future investigations should incorporate an inert activity or usual-training comparator to isolate time and season-related influences. Second, the cohort was predominantly male (89%), limiting the generalisability of conclusions, and statistical power for sex-stratified analyses was insufficient. Larger, sex-balanced samples are warranted to test for potential dimorphic redox adaptations.

This study has additional limitations as the level of superoxide in plasma, which can be reduced by SOD, was not investigated in the current study. Catalase and other antioxidants were also not included in the investigation. It did not allow a more complex assessment of antioxidative system activity [2]. One of possible factors affecting the results is the interval between the end of long-term training and the collection of peripheral blood. It was performed the next morning after the long-term training and included about 12 h of rest. Despite this, we recommend detecting OS markers in plasma with markers of cell damage for the objective assessment of the health status of participants at the pre- and post-training phases.

## 5. Conclusions

The decrease in oxidants in plasma and OS index despite the discordant change in antioxidative system components reflects the complex adaptation processes to long-term multi-stressor training. These changes include upregulation of the glutathione system with simultaneously decreasing SOD activity. Additionally, we did not reveal any evidence of cell damage after the training that point to successful adaptation for long-time multi-stressor in participants.

## Figures and Tables

**Table 1:** H_2_O_2_—hydrogen peroxide; SOD—superoxide dismutase activity; GSSG—oxidative glutathione; GSH—reduced glutathione; OS index—oxidative stress index; 8-OHdG—8-hydroxy-2-deoxyguanosines; MDA—malondialdehyde; opt. d. U.—optical density units; IQR—interquartile interval.

Parameters	Baseline	After the Training	Wilcoxon Test	
	Median (IQR)	Median (IQR)	W	*p*
H_2_O_2_, opt. d. U.	0.10 (0.07; 0.15)	0.08 (0.07; 0.10)	1528.00	0.015
SOD, opt. d. U.	0.62 (0.47; 0.70)	0.50 (0.46; 0.58)	1630.00	0.002
GSSG, opt. d. U.	0.98 (0.90; 1.10)	1.29 (1.19; 1.53)	56.00	<0.001
GSH, opt. d. U.	0.56 (0.50; 0.64)	1.54 (1.27; 1.76)	1.00	<0.001
OS index	1.78 (1.63; 1.93)	0.95 (0.75; 1.15)	2302.00	<0.001
8-OHdG, ng/mL	1.95 (1.74; 2.27)	1.50 (0.96; 1.93)	2067.00	<0.001
MDA, opt. d. U.	0.14 (0.10; 0.15)	0.07 (0.06; 0.09)	2304.00	<0.001
Myoglobin, ng/mL	23.00 (18.47; 28.27)	23.73 (18.53; 33.07)	1255.50	0.603

## Data Availability

The raw data supporting the conclusions of this article will be made available by the authors on request.

## Abbreviations

The following abbreviations are used in this manuscript:

8-OHdG	8-hydroxy-2-deoxyguanosine
BMI	Body Mass Index
CAT	Catalase
CI	Confidence Interval
EDTA	Ethylenediaminetetraacetic Acid
ELISA	Enzyme-Linked Immunosorbent Assay
GSH	Reduced Glutathione
GSSG	Oxidised Glutathione
H_2_O_2_	Hydrogen Peroxide
IQR	Interquartile Interval
MDA	Malondialdehyde
OS	Oxidative Stress
OS index	Oxidative Stress Index
ROS	Reactive Oxygen Species
SPSS	Statistical Package for the Social Sciences
SOD	Superoxide Dismutase
TAC	Total Antioxidant Capacity
opt. d. U.	Optical Density Units
